# Comparison of Three Types of Continent Urinary Diversions in a Single Center

**DOI:** 10.1100/tsw.2004.59

**Published:** 2004-06-07

**Authors:** Cengiz Girgin, Akif Sezer, Kutan Ozer, Huseyin Tarhan, Ahmet Bolukbasi, Gozen Gurel

**Affiliations:** Ataturk Training Hospital Urology Department, Izmir, Turkey

## Abstract

The results including the complication and continence rates for 3 types of continent urinary diversion were evaluated. From 1992 to 1998 we performed 58 continent urinary diversions after radical cystectomy for invasive transitional cell carcinoma (TCC) of the urinary bladder. All three types of continent diversions and ileal loop procedures were discussed and patient preferences were determined. The patient preference rate for continent urinary diversion was 96.6%, and half of these patients wanted to be completely dry. Mean age of the patients was 58.2 years. Of the 58 patients, 9 (15.5%) had a Kock pouch, 15 (25.8%) had a Kock neobladder and 34 (58.6%) had sigmoidorectal pouch (Mainz-II pouch). Early and late complication rates of the three different continent diversions were evaluated. The number of complications, such as urine leakage, pyelonephritis, hydronephrosis, reflux and stone formation, were similar in all three types of diversions. Two (5.9%) Mainz pouch II patients who had stopped oral alkalinization demonstrated severe hyperchloremic acidosis. Spontaneous pouch rupture occurred in 1 of the Kock pouches. Reoperation rates were higher with the Kock pouch and Kock neobladder cases. Daytime continence rates for the Kock pouch, Kock neobladder and Mainz II pouch were 77.7%, 86.7% and 100% respectively. Even though complete dryness may not be achieved in every patient, orthotopic bladder substitution appears to be the best choice after radical cystectomy. Although it carries the risk of life-long oral alkalinization therapy, the Mainz pouch II is associated with an excellent continence rate and may be a good alternative for patients who desire to be dry.

## INTRODUCTION

Various forms of continent urinary diversions are available for reconstruction of the lower urinary tract after radical cystectomy for invasive TCC. Continent cutaneous stomal reservoirs, orthotopic bladder substitutes and sigmoidorectal pouches are the most common forms of continent urinary diversions employed today. Continent stomal urinary reservoirs are replacing the ileal conduit (Bricker conduit) as the standard procedure for urinary diversion. Castagnola et al. reported that continent stomal diversions demonstrated a higher rate (97%) of patient satisfaction and a lower incidence of stomal problems than ileal conduits (1). Even though high patient satisfaction rates have been achieved, continent stomal urinary reservoirs generally had higher incidence rates of early and late complications and reoperation rates (2,3). At most centers, orthotopic bladder substitution is the first choice for urinary diversion in men, with satisfactory continence rates reported as 65-98% during the daytime and 37-87% at night (4). Ureterosigmoidostomy was the first form of continent urinary diversion, but it had higher incidences of complications such as hyperchloremic acidosis, recurrent pyelonephritis and loss of renal function. The technique of ureterosigmoidostomy has been modified by some authors in recent years and a low pressure sigmoidorectum pouch (Mainz-II pouch) has been created by detubularization of the rectosigmoid. Atta et al. reported high daytime and nighttime continence rates and a low incidence of complications with the Mainz pouch II (5,6). In this study, we evaluated patient preferences and results using three types of continent urinary diversions in our department between 1992 and 1998.

## PATIENTS AND METHOD

Between 1992 and 1998, 58 continent urinary diversions were performed after radical cystectomy for TCC of the bladder in our department. The mean age of the patients was 58.2 (range 45 to 65) years. There were 55 (94.8 %) male and 3 (5.2%) female patients. All patients had muscle invasive TCC demonstrated histologically in TUR biopsies. Chest X-rays, blood chemistry analyses, IVP and abdominal CT were obtained prior to radical cystectomy to rule out metastatic disease. A creatinine level above 1.5mg/dl was the exclusion criterion for continent urinary diversion. Anal sphincter integrity was measured by 3 hours of continence after a 400ml. water infusion into the rectum for the Mainz pouch II cases. Preoperative evaluation in these patients also included radiological imaging of the colon to rule out diverticulosis or polyps. In all Mainz pouch II cases, prophylactic oral alkalinization therapy was used. The mean follow up period was 23.5 months (range 2 to 69).

The patient selection criteria for the 3 types of continent urinary diversions included patient preference and the stage of disease, as follows:

A When disease was confined to the bladder without urethral or prostatic stromal involvement, a Kock neobladder was performed.
B When disease was confined to the bladder with high risk of urethral or pelvic recurrence, a Kock pouch was performed.
C When disease was confined to the bladder, or local invasive disease and/or prostatic stromal invasion was present without nodal metastasis, or the patients were female, a Mainz pouch II procedure was performed.

Complication and continence rates of the three types of continent urinary diversions were discussed with the patients and their preferences were evaluated. The patientís preferred procedure was performed, if the stage of the primary disease was suitable for that type of diversion.

Kock pouch and Kock neobladder procedures were performed by the modified technique described by Skinner in 1989 (7) and the Mainz II pouch was performed by the technique described by Fisch and Hohenfeller in 1991 (8). In 58 cases, 34 Mainz pouch II procedures, 15 Kock neobladders and 9 Kock pouch procedures were performed. The mean follow-up period was 18.4 months (range 6 to 40) for the Mainz II pouch procedure, 16.8 months (range 2 to 48) for the Kock neobladders, and 35 months (range 6 to 69) for the Kock pouches. The follow-up protocols are listed in Table 1.

**TABLE 1**
**Follow-up schedule of the patients. Blood chemistry included urea, creatine, Na, K, Cl, Ca, blood gases and pH**

| Months after surgery | Blood Chem. | Chest X-Ray/IVP | USG. | Abdominal CT | Urethral washing cytology |
|---|---|---|---|---|---|
| 3 | + | +/- | + | - | + |
| 6 | + | +/+ | + | + | + |
| 9 | + | +/- | + | - | + |
| 12 | + | +/+ | + | + | + |
| 18 | + | +/- | + | + | + |
| 24 | + | +/- | + | + | + |
| 36 | + | +/- | + | + | + |

## RESULTS

Only 2 of the 60 patients (3.4%) preferred an ileal loop procedure. Among the remaining 58 patients, 19 (32.7%) deferred the decision to the surgeon.There were no operative mortalities in the 58 cases of radical cystectomy and continent urinary diversion. Pathological stages after radical cystectomy were 24 (41.4%) pT2, 12 (20.7%) pT3a, 17 (29.3%) pT3b and 5 (8.6%) with N1 disease. Patients having nodal metastases required 4 courses of MVAC chemotherapy. One Kock pouch and one Mainz pouch II patient (3.4%) had urethral recurrence 14 and 15 months after radical cystectomy, respectively. Both cases underwent urethrectomy and required 4 courses of MVAC chemotherapy. Of these two patients, the Kock pouch patient died because of brain metastasis 6 months after chemotherapy. In the mean follow-up period of 23.5 months, 14 (24.1%) patients died because of their primary disease. Of these 14 cases, 5 had a Kock pouch, 4 had a Kock neobladder and 5 had a Mainz pouch II type of diversion. There were no continent diversion related deaths.The mean operation time was 4 hours for the Kock pouch and Kock neobladder procedures and 1.5 hours for the Mainz pouch II procedures.

Early reoperation rates for Mainz pouch II, Kock neobladder and Kock pouch procedures were 2.9%, 6.7% and 11.1% respectively. The Mainz pouch II and Kock neobladder cases were reoperated due to urine leakage from the ureterointestinal anastamosis. One patient with a Kock pouch was reoperated due to urine leakage from the pouch. Early postoperative complications for the three types of continent diversions are listed in Table 2.

In 1 patient (11.1%) with a Kock pouch a spontaneous rupture occurred twice because of delayed catheterization. The extraperitoneal ruptures healed with prolonged catheter drainage and without surgical intervention. Two cases (22.2%) with a Kock pouch were reoperated due to difficulty in self - catheterization. Mild metabolic acidosis compensated by oral alkalinization occurred in 24 patients (70.6%) with a Mainz pouch II. Only 2 patients (5.9%) which stopped oral alkalinization showed severe hyperchloremic acidosis necessitating hospital admission. Other complications, such as pyelonephritis, reflux and hydronephrosis are listed in Table 3. None of the continent diversion cases showed new stone formation or vitamin B12 deficiency.

Patients using more than one pad per day were considered incontinent. Day-time continence rates in the third postoperative month were as follows: 100% for the Mainz pouch II, 86.7% for the Kock neobladder and 77.7% for the Kock pouch. One case of Mainz pouch II and 3 cases of Kock neobladders had nocturnal enuresis. Nighttime continence rates were 97% and 80% respectively in the Mainz pouch II and Kock neobladder cases. All Kock neobladder patients voided spontaneously with less than 50 ml of residual urine. Only one patient needed intermittent self-catheterization.

**TABLE 2**
**Early Postoperative Complications of Three Types of Continent Urinary Diversions**

| **Procedure**<br><br>**No. of Patients** | **Intestinal asastomosis related comp.**<br><br>**n (%)** | **Urine leakage from pouch**<br><br>**n (%)** | **Urine leakage from ureter anastomosis**<br><br>**n (%)** | **Urosepsis**<br><br>**n (%)** | **Mucus causing catheter occlusion**<br><br>**n (%)** | **Wound infection**<br><br>**n (%)** | **Wound dehiscence**<br><br>**n (%)** |
|---|---|---|---|---|---|---|---|
| Mainz pouch II<br><br>n=34 | 0 | 0 | 1 (2.9%) | 1 (2.9%) | 0 | 2 (5.9%) | 1 (2.9%) |
| Hemi-Kock<br><br>n=15 | 0 | 0 | 1 (6.7%) | 0 | 3 (20%) | 1 (6.7%) | 1 (6.7%) |
| Kock pouch<br><br>n=9 | 0 | 1 (11.1%) | 0 | 0 | 3 (33.3%) | 1 (11.1%) | 0 |

**TABLE 3**
**Late complication rates of three types of continent urinary diversions**

| **Procedure**<br><br>**No. of Patients** | **Pyelonephritis**<br><br>**n (%)** | **Severe metabolic acidosis**<br><br>**n (%)** | **Difficult catheterisation**<br><br>**n (%)** | **Hydronephrosis**<br><br>**n (%)** | **Reflux**<br><br>**n (%)** | **Reoperation**<br><br>**n (%)** |
|---|---|---|---|---|---|---|
| Mainz pouch II<br><br>n=34 | 1 (2.9%) | 2 (5.8%) | - | 1 (2.9%) | 0 | 0 |
| Hemi-Kock<br><br>n=15 | 1 (6.7%) | 0 | 0 | 1 (6.7%) | 2 (13.4%) | 0 |
| Kock pouch<br><br>n=9 | 1 (11.1%) | 1 (11.1%) | 2 (22.2%) | 0 | 2 (22.2%) | 2 (22.2%) |

## DISCUSSION

Continent reconstruction of the lower urinary tract for severe bladder dysfunction or bladder cancer has been employed widely in recent years. Continent cutaneous stomal reservoirs, orthotopic bladder substitutes and sigmoidorectal pouches are the most common forms of continent urinary diversions employed today. When deciding which of these procedures to recommend to the patient, one must consider the functional outcome, the potential short and long-term complications and the physical and psychosocial readjustments required following surgery (9).

## Kock pouch

The Kock pouch procedure has been associated with high reoperation rates. Using the technique described by Skinner et al (7), we obtained 77.7% daytime and nighttime continence rates in a small group of patients, but the overall early and late complication rates were high (Table 2 and 3). Also, 33.3% of the patients were reoperated due to either urine leakage from the pouch (11.1%), or due to difficulty in catheterization (22.2%). In other series, continence rates with the Kock pouch ranged between 76 to 90% and approximately one-third of the patients needed to be reoperated (7,10,11). Highly motivated patients are essential for the Kock pouch type of diversion, because delay in catheterization may lead to spontaneous pouch rupture, as seen in one of our cases. Our experience based on this small group of patients showed that the Kock pouch type of diversion has a steep learning curve and may be associated with significant complications even when the procedure is performed by experienced surgeons.

## Kock neobladder

There is an increasing interest in using orthotopic bladder substitutes or neobladders for reconstruction. However, these procedures are not suitable for every patient, especially for those with cancer who have a high risk of urethral recurrence. Different segments of the gastro-intestinal tract can be used for construction of the orthotopic bladder. An efficient antireflux mechanism and a reasonable pouch volume with low pressures may be achieved with the Kock neobladder. In our patients, continence was achieved in 86.7% of patients during the daytime and 80% of patients during the nighttime. The reoperation rate was 6.7%. The main long term complication of this type of diversion was related to the afferent anti-reflux nipple valve. Reflux in 2 (13.4%) patients resulted in pyelonephritis in one case and hydronephrosis in the other.

The continence rates in the literature, for different types of ileal neobladders, have ranged between 85 to 92% during the daytime and 55.5 to 85% at nighttime (12,13,14,15,16). This wide range of nighttime continence rates is the result of differing definitions of continence by different authors. Elmajian et al. reported 87% and 86% good or satisfactory daytime and nighttime continence, respectively, with pouch related early and late complication rates of 7.2% and 11.6%, respectively. They also reported pouch related reoperation rates of 1.4% in a large series of Kock neobladder cases (17). The main advantage of orthotopic neobladders is the ablility to void via the urethra, which is encouraging to patients undergoing a radical cystectomy operation.

## Mainz pouch II

Good results and high continence rates have been achieved with the sigmoidorectal pouch (ranging from 94.5 to 100%) by several groups (5,6,18,19,20). Our studies with 34 Mainz pouch II patients showed a 100% daytime continence rate, and only one patient had nocturnal enuresis. The main disadvantage of the Mainz pouch II procedure was the presence of metabolic acidosis, which can be managed by oral alkalinization therapy. Gilja reported the presence of hyperchloremic acidosis in his patients, and 65% needed oral alkalinization (6). We used prophylactic oral alkalinization in all patients, and in 2 cases (5.9%), stopping prophylactic therapy resulted in severe acidosis necessitating hospital admission. The Mainz pouch II continent diversion procedure has the advantage of a short operation period (mean 90 minutes) and a low incidence of early or late complications. However, follow-up is necessary in order to determine if there is any possibility for intestinal malignancies long term.

All three types of continent diversions performed in our center were associated with satisfactory continence rates. The Kock pouch procedure had the highest reoperation and complication rates in our series. We believe the Mainz pouch II or ileal neobladder diversion procedure should be considered preferable for patients who are candidates for continent diversion. Mansson commented in his editorial paper that ìin light of enthusiasm for specific methods with excellent results in experienced hands, urinary reconstruction should be tailored more to meet the needs of the patient, who should be able to choose between all available methodsî (21). Patient preference should play a significant role in the selection of the type of continence diversion procedure. Several different continent diversion procedures may be suitable for any individual patient with varying advantages and disadvantages. These alternatives and their parameters should be discussed clearly with the patient. One-third of patients in our study left the decision to the surgeon.

## CONCLUSION

Approximately 97% of patients undergoing radical cystectomy in our study preferred a continent type of diversion when it was possible, and patients believed they would have a better quality of life as a result. Also, patient acceptance for radical cystectomy was made easier when there was a possibility of a continent type of diversion rather than an ileal loop. Half of the patients wanted to be completely dry and only 15.5% wanted to urinate through the urethra. Even though complete dryness may not be achieved in every patient, orthotopic bladder substitution appears to be preferable after radical cystectomy. Although the Mainz pouch II carries the risk of life-long oral alkalinization therapy, it is a good alternative for patients who prefer to be dry.
